# Variable Presentations of Emphysematous Liver Abscesses: Experiences From Four Cases at a Tertiary Care Center

**DOI:** 10.7759/cureus.69200

**Published:** 2024-09-11

**Authors:** Sukesh KS, Abhinav A Sonkar, Akshay Anand, Manish Kumar Agarwal, Kushagra Gaurav

**Affiliations:** 1 General Surgery, King George's Medical University, Lucknow, IND; 2 Surgery, King George's Medical University, Lucknow, IND

**Keywords:** emphysematous liver abscess, gas forming bacteria, gas under diaphragm, klebsiella, : liver abscess, pcd- percuateneous catheter drainage

## Abstract

Emphysematous liver abscesses usually present with fever and abdominal pain with radiological investigations showing air inside the abscess cavity and biochemical parameters suggestive of sepsis. This is a condition that needs urgent intervention, but it can present with variable presentations and the gas under the right dome of the diaphragm makes the diagnosis in dilemma, confusing it with hollow viscous perforation. Hereby we present a case series of variable presentations of emphysematous liver abscesses, successfully managed by timely intervention.

## Introduction

Emphysematous liver abscesses are always accompanied by gas formation and hence are also known as gas-forming pyogenic liver abscesses [1]. Management of acute emphysematous liver abscess is urgent external drainage of the abscess cavity [1], but gas under the right hemidiaphragm on radiological imaging can pose a serious diagnostic dilemma and can misguide surgeons, leading to them performing unnecessary exploratory laparotomy. Also, the variable presentations of the condition make it a diagnostic challenge.

## Case presentation

Case 1

**Figure 1:**
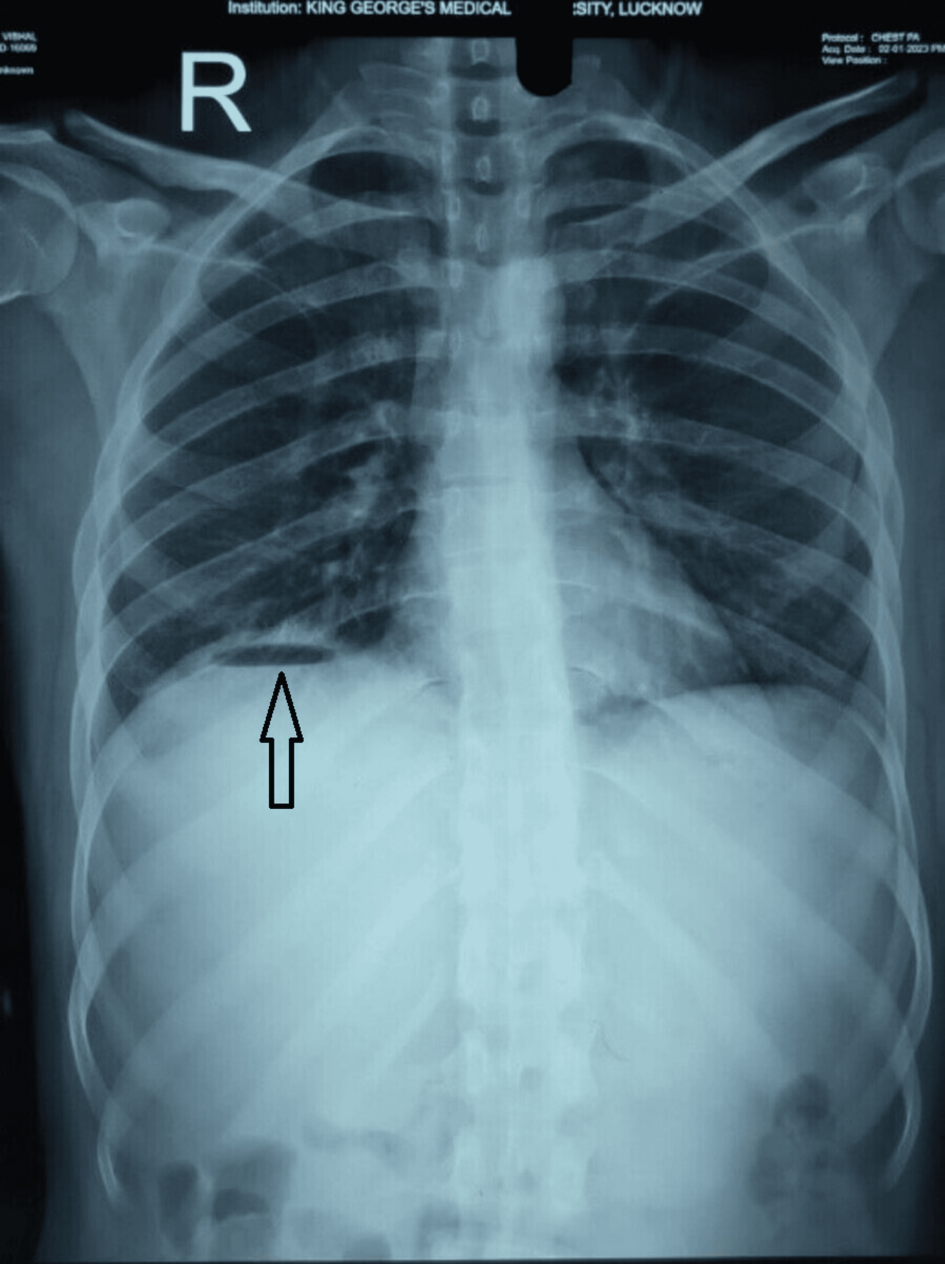
Abdominal X-ray showing gas under the right diaphragm (arrow)

A 22-year-old male patient with no comorbidities and a history of occasional alcohol intake came to the surgical outpatient department with a history of on and off right upper quadrant pain for seven days associated with mild fever; an outside chest X-ray showed gas under the right hemidiaphragm. On examination, the patient was afebrile, hemodynamically stable and the abdomen was soft and non-tender. Repeat chest X-ray revealed a similar finding (Figure 1) and ultrasonography (USG) of the whole abdomen revealed a liver abscess in the right lobe of the liver.

**Figure 2:**
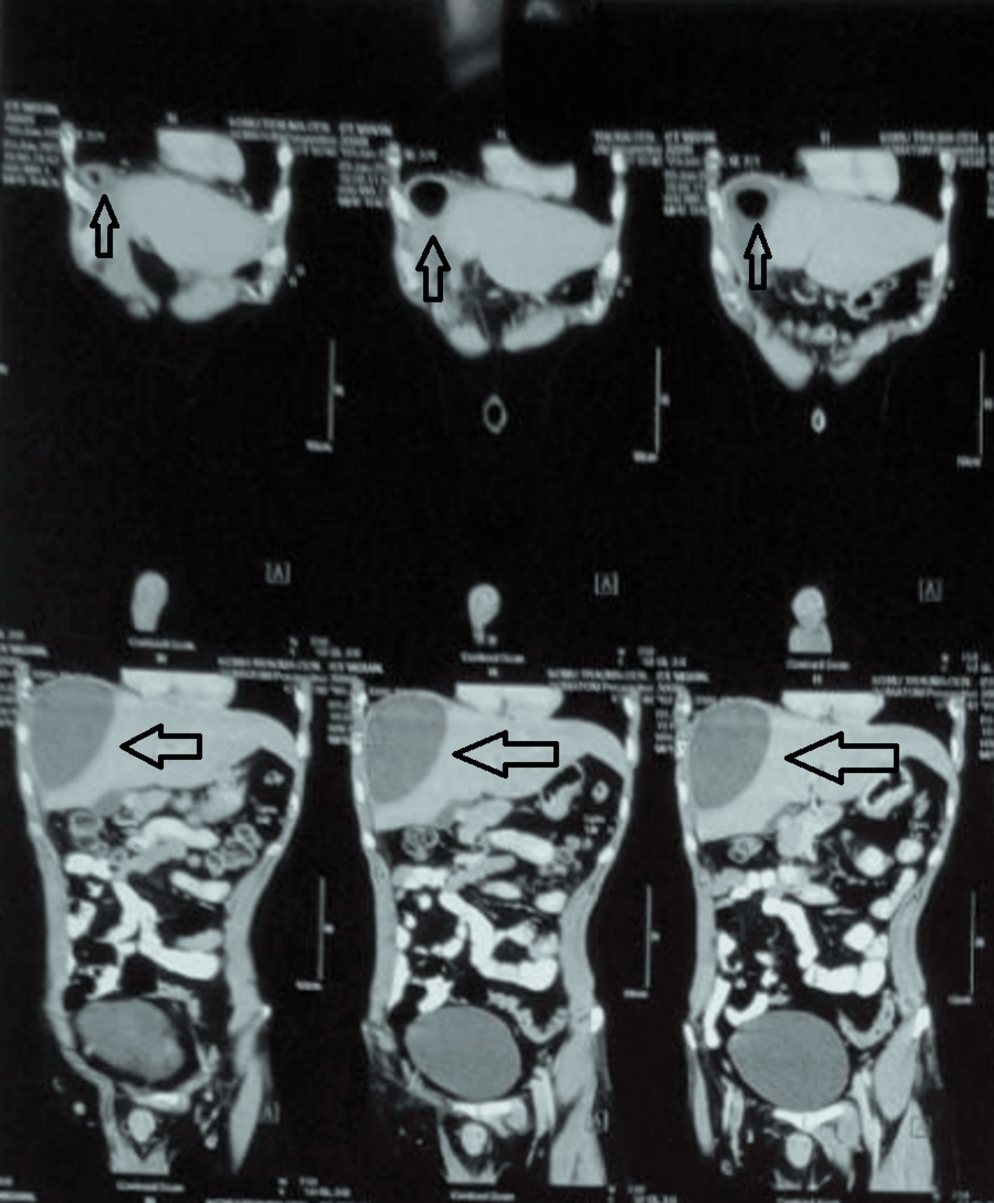
CECT (coronal) of patient 1 showing hypodense liver abscess with emphysematous changes (arrows) CECT: contrast-enhanced computerized tomography

**Figure 3:**
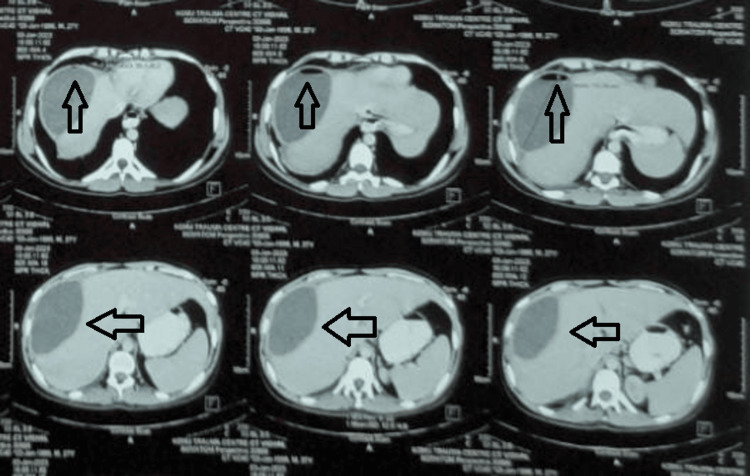
CECT (axial) of patient 1 showing hypodense liver abscess with emphysematous changes (arrows) CECT: contrast-enhanced computerized tomography

Contrast-enhanced computerized tomography (CECT) abdomen and thorax was done which was suggestive of a single hypodense space-occupying lesion with air-fluid levels visible inside the lesions in the right lobe of the liver (Figures 2, 3). Patients’ biochemical parameters were within normal limits and were managed by external drainage with pigtail catheter insertion in the liver abscess cavity with an output of 100 ml of pus. Cultures revealed *Klebsiella pneumoniae* as the causative agent. The patient was treated with IV antibiotics covering gram-negative and anaerobic spectrum and was discharged after seven days after repeat ultrasonography. On follow-up, the patient was doing well and the drain was removed after 10 days.

Case 2

**Figure 4:**
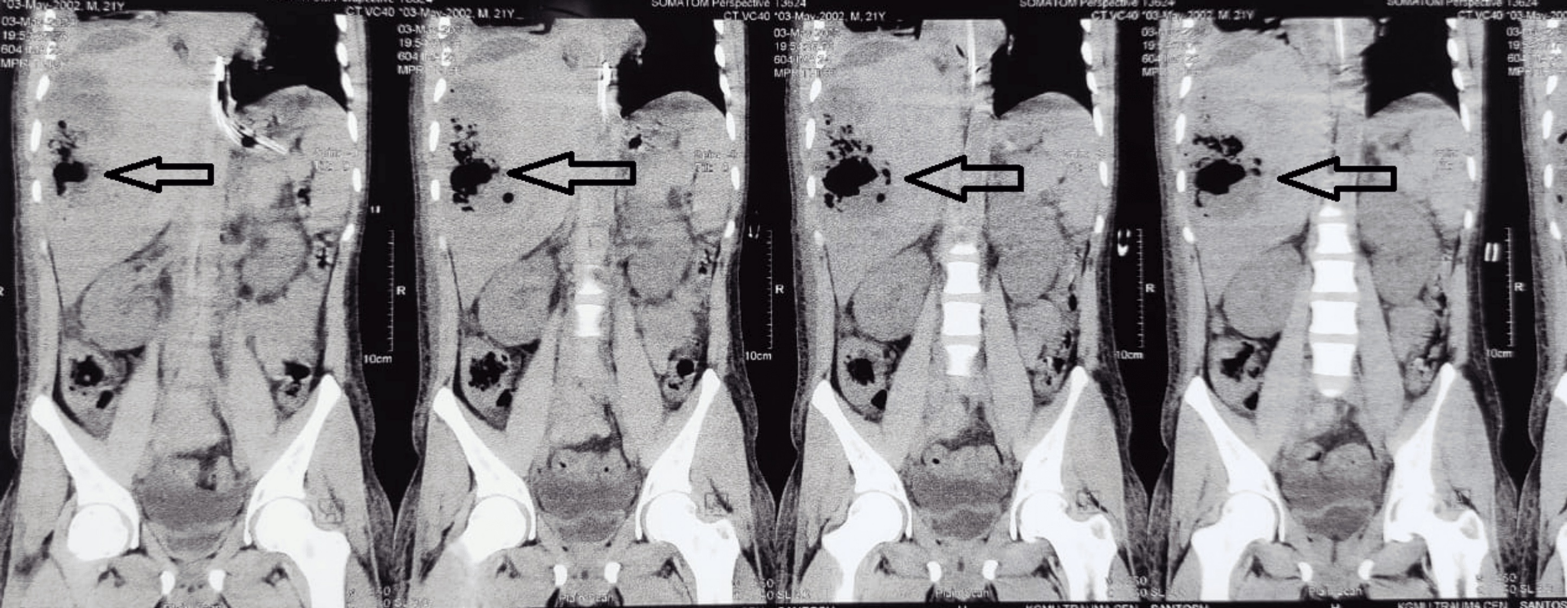
NCCT (coronal) of patient 2 showing hypodense liver abscess with emphysematous changes (arrows) NCCT: non-contrast computerized tomography

**Figure 5:**
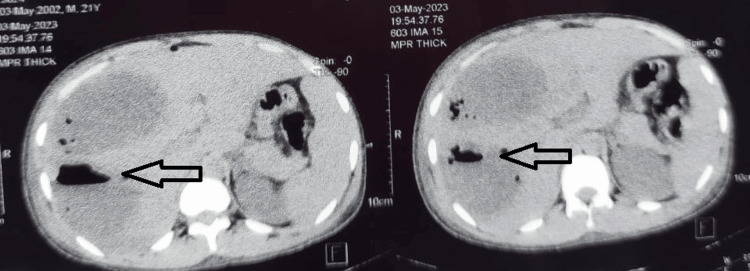
NCCT (axial) of patient 2 showing hypodense liver abscess with emphysematous changes (arrows) NCCT: non-contrast computerized tomography

An 18-year-old male alcoholic and smoker with no other comorbidities came to the surgical emergency with complaints of severe abdominal pain and high-grade fever with chills for seven days. On examination, the patient was febrile with tachycardia, hypotension, and abdominal tenderness with guarding and rigidity. X-ray erect abdomen was not suggestive of any pathology. Ultrasonography revealed multiple abscesses in the right lobe of the liver with no intra-abdominal collection. The patient’s biochemical parameters were suggestive of MODS (Multiple Organ Dysfunction Syndrome). Hemoglobin was 8g/dl; total leucocyte count was 28000 cells/mm3 with 85% polymorphs; platelet count was 35000 cells/mm3; urea was 180 mg/dl; creatinine was 3.8 mg/dl; INR (international normalized ration) was 3.7 with prothrombin time of 36; liver function tests were bilirubin of 3 mg/dl, alkaline phosphatase of 400 IU/dl, SGOT (serum glutamic oxaloacetic transaminase) of 120 IU/dl and SGPT (serum glutamic pyruvic transaminase) of 110 IU/dl. The patient was resuscitated and a non-contrast CT (NCCT) abdomen was done which revealed multiple hypodense space-occupying lesions along with homogenous collection in the liver parenchyma with air noted inside the cavities in the right lobe of the liver (Figures 4, 5). After initial resuscitation patient was managed by external drainage with multiple pigtail catheter insertions in the liver abscess cavity and empirical broad-spectrum antibiotics. Cultures revealed *Klebsiella pneumoniae* as the causative agent. The patient's condition improved after drainage. The patient was treated with IV antibiotics covering gram-negative and anaerobic spectrum and was discharged after 14 days after repeat ultrasonography. On follow-up, the patient was doing well and the drain was removed after 20 days.

Case 3

**Figure 6:**
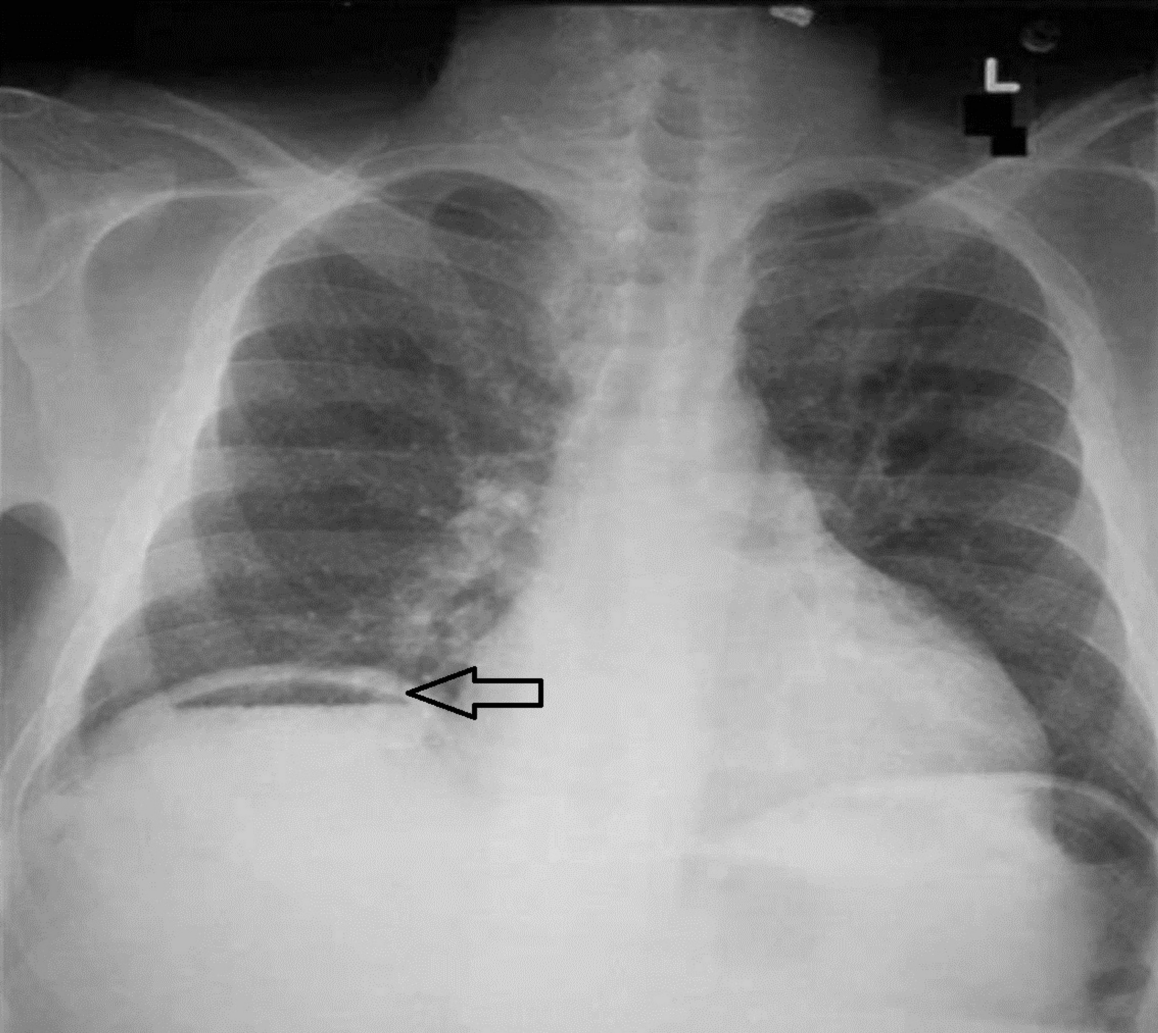
Abdominal X-ray of patient 3 showing gas under the diaphragm (arrow)

A 50-year-old homemaker, a known diabetic with uncontrolled sugars presented to the surgical emergency with complaints of intermittent fever with chills and right upper quadrant pain for 10 days. On examination, the patient was afebrile, hemodynamically stable and the abdomen was soft with right hypochondrial tenderness. An erect X-ray of the abdomen was suggestive of gas under the right hemidiaphragm (Figure 6) and ultrasonography revealed two liver abscesses in the right lobe of the liver.

**Figure 7:**
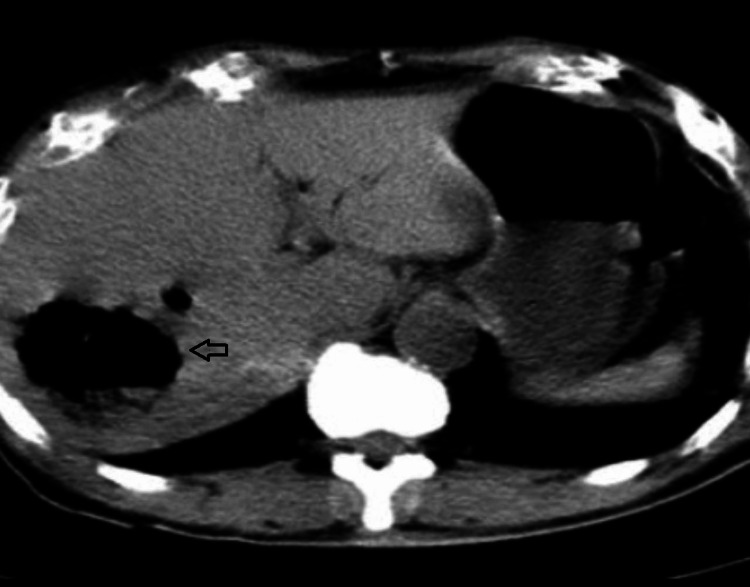
CECT (axial) of patient 3 showing hypodense liver abscess with emphysematous changes (arrow) CECT: contrast-enhanced computerized tomography

CECT of the whole abdomen was suggestive of two large hypodense space-occupying lesions along with homogenous collection in the liver parenchyma, involving segments 8, 2, and 3 of the liver; air was also noted, with air-fluid levels visible inside the hypodense lesions (Figure 7). The patient was managed by external drainage with pigtail catheter insertion in the liver abscess cavity and glycemic control. The patient was treated with IV antibiotics covering gram-negative and anaerobic spectrum and was discharged after 10 days after repeat ultrasonography. On follow-up, the patient was doing well and the drain was removed after 14 days.

Case 4

**Figure 8:**
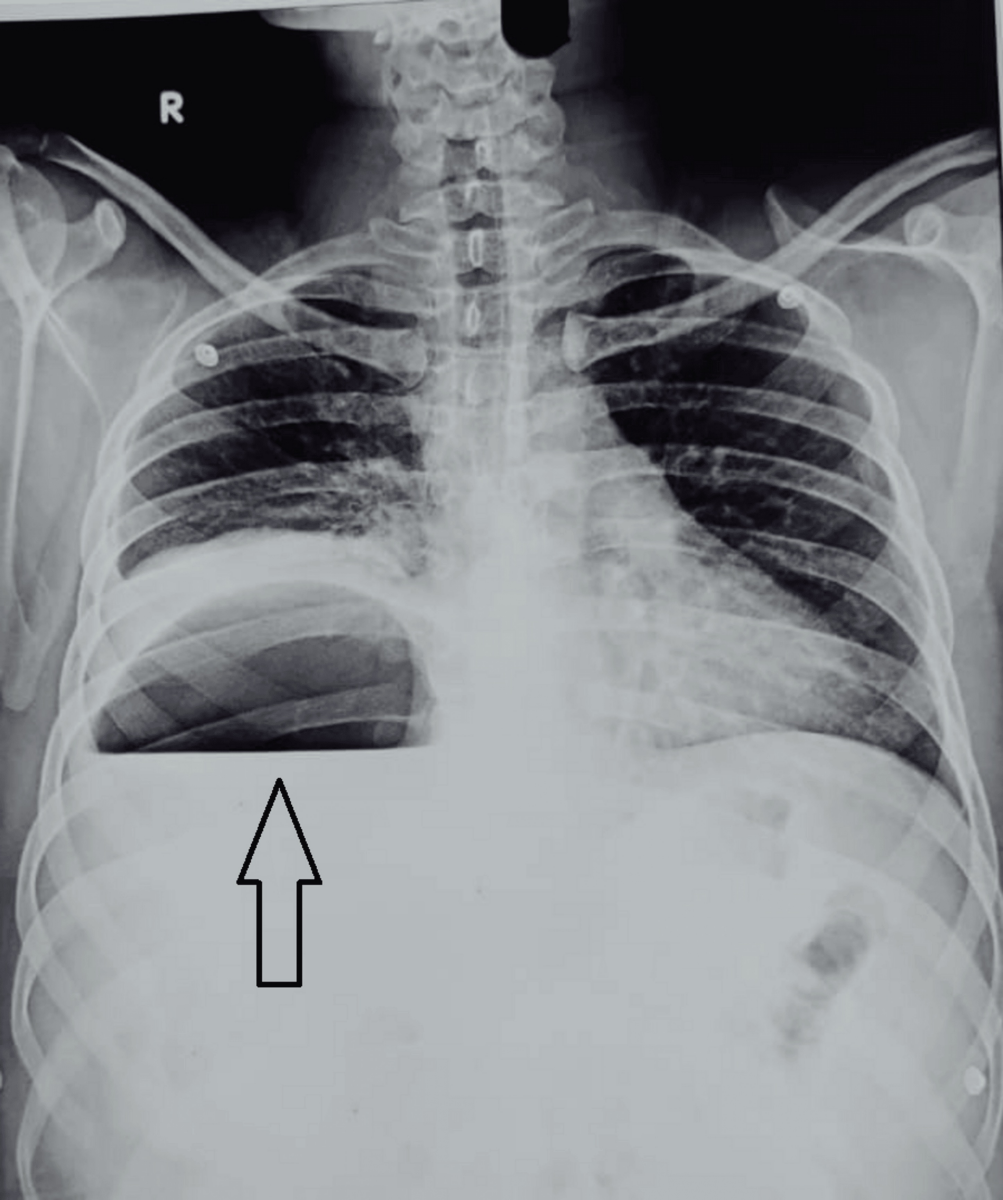
Abdominal X-ray of patient 4 showing gas under the right dome of the diaphragm (arrow)

A 32-year-old male who underwent laparoscopic cholecystectomy one month back, presented with complaints of abdominal pain and on-and-off fever for seven days; the patient was hemodynamically stable with localized tenderness in the right hypochondrium. X-ray abdomen showed gas under the right dome of the diaphragm (Figure 8) and USG showed a 400 ml hypoechoic lesion in segment 7 liver suggestive of liver abscess.

**Figure 9:**
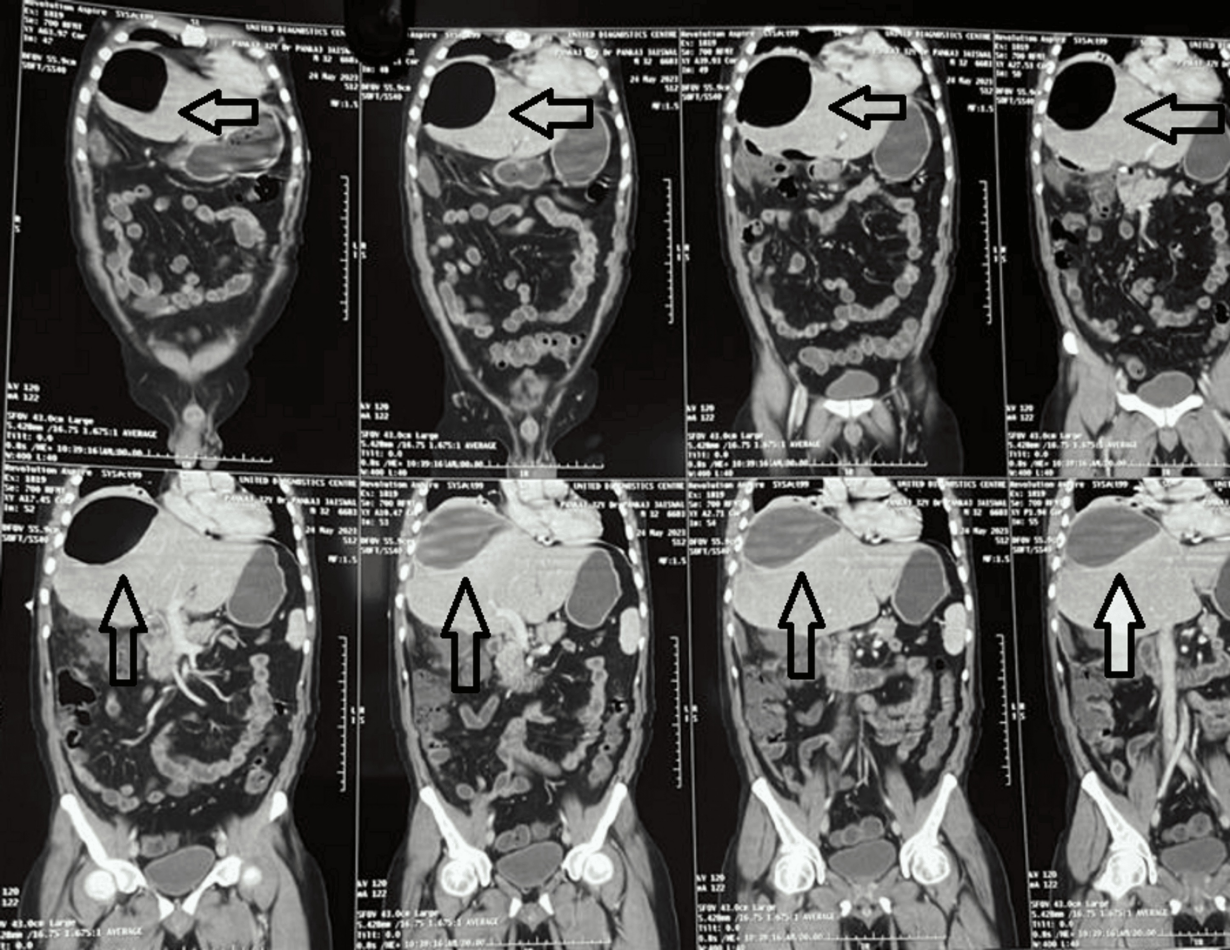
CECT (coronal) of patient 4 showing hypodense liver abscess with emphysematous changes (arrows) CECT: contrast-enhanced computerized tomography

**Figure 10:**
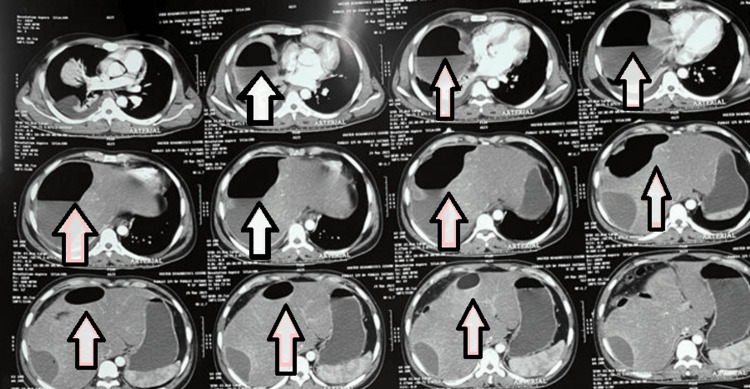
CECT (axial) of patient 5 showing hypodense liver abscess with emphysematous changes (arrows) CECT: contrast-enhanced computerized tomography

CECT abdomen was suggestive of a hypointense lesion with specs of air in segment 7 of the liver suggestive of emphysematous liver abscess (Figures 9, 10). The patient was managed by percutaneous drainage of the liver abscess and the pus culture was sterile. The patient was treated with IV antibiotics covering gram-negative and anaerobic spectrum and was discharged nine days after repeat ultrasonography. On follow-up, the patient was doing well and the drain was removed after 14 days.

## Discussion

Emphysematous liver abscesses are typically characterized by gas formation within the abscess cavity [1]. Of all bacterial liver abscesses, 6-24% are emphysematous in nature [2], and Asia reports the maximum number of cases of emphysematous liver abscesses, with Taiwan reporting the maximum incidence [3]. The most common species causing it in Asian countries is *Klebsiella pneumoniae* (70% of cases) [4]. It has a high mortality rate of 27% [3]. Diabetics and those patients with poor glycemic control (HbA1c>=8%) have an increased risk of developing emphysematous liver abscesses because hyperglycemia is a favorable condition for gas formation due to glucose metabolism by the mixed acid fermentation pathway, which leads to the production of formic acid, which that gets converted to formate hydrogenylase after excessive accumulation (pH<=6), which breaks down further into carbon dioxide and hydrogen [3]. The production of hydrogen is the hallmark feature of the mixed acid fermentation pathway because it is not produced in any of the other five fermentation pathways [3]. In a study by Lee HL et al., analyses of gas samples from five patients with gas-forming liver abscess due to *Klebsiella pneumoniae* showed the following composition: nitrogen, 65.8 to 78.1%; oxygen, 1.2 to 7.3%; carbon dioxide, 5.4 to 14.8%; and hydrogen, 9.0 to 18.3% [3].

Emphysematous liver abscesses often become prone to rupture, due to mass tissue damage and a rise in internal pressure due to the formation of gases [5]. Abdominal ultrasonography, simple radiography, and other imaging techniques might prove to be helpful for diagnosis, but CT is the diagnostic tool of choice for accurate detection of gas within abscesses [6]. Treatment is mainly by percutaneous abscess drainage, along with antibiotic therapy and glycaemic control [7].

In our cases, three hemodynamically stable patients with no clinical findings presented with gas under the right hemidiaphragm, as seen on radiological imaging, which was a diagnostic dilemma, and can easily misguide the surgeon as he/she might end up performing an unnecessary laparotomy in a search of a hollow viscus perforation in the abdomen and was diagnosed to be emphysematous liver abscess after CECT as the clinical and X-ray findings were contradictory. Contrastingly another patient with signs and symptoms of acute abdomen with multiorgan dysfunction had no gas under the right hemidiaphragm but had emphysematous liver abscesses.

In a similar case reported by Maliyakkal AM et al., the patient presented with acute abdominal pain, with gas under the diaphragm, as seen in imaging, which was attributed to a ruptured hepatic abscess caused by *Klebsiella pneumoniae*. The patient was managed by ultrasound-guided percutaneous aspiration and drain insertion, coupled with antibiotic therapy and supportive treatment. Laparotomy was not performed [8]. Whereas, in another case reported by Shiba H et al., a woman presented with high fever, followed by acute abdominal pain a day later. CT showed an abscess with gas in the lateral segment of the liver, with spontaneous pneumoperitoneum. The patient had to undergo an emergency lateral segment hepatectomy and the culture from the liver tissue, abscess, and blood showed *Klebsiella pneumoniae *[9].

## Conclusions

Emphysematous liver abscesses have variable presentations. It can present with or without abdominal pain, features of sepsis, and gas under the diaphragm usually in a patient with other comorbidities. The presence of gas under the diaphragm may prompt for laparotomy. Ultrasonography and contrast-enhanced computerized tomography with clinical history and examination will help in proper diagnosis. Thus, proper evaluation will avoid unnecessary laparotomy, and timely and less invasive interventions like percutaneous drainage are life-saving.
